# Landscape‐scale deforestation decreases gene flow distance of a keystone tropical palm, *Euterpe edulis* Mart (Arecaceae)

**DOI:** 10.1002/ece3.2341

**Published:** 2016-08-24

**Authors:** Alesandro S. Santos, Eliana Cazetta, Pavel Dodonov, Deborah Faria, Fernanda A. Gaiotto

**Affiliations:** ^1^ Pós‐Graduação em Ecologia e Conservação da BiodiversidadeUniversidade Estadual de Santa CruzRodovia Ilhéus‐Itabuna, km 16IlhéusCEP 45662‐900Brazil; ^2^ Laboratório de Ecologia Aplicada à ConservaçãoDepartamento de Ciências BiológicasUniversidade Estadual de Santa CruzRodovia Ilhéus‐Itabuna, km 16IlhéusCEP 45662‐900Brazil; ^3^ Laboratório de Marcadores MolecularesCentro de Biotecnologia e GenéticaUniversidade Estadual de Santa CruzRodovia Ilhéus‐Itabuna, km 16IlhéusCEP 45662‐900Brazil

**Keywords:** Conservation, functional connectivity, landscape genetics, tropical forest

## Abstract

Habitat loss represents one of the main threats to tropical forests, which have reached extremely high rates of species extinction. Forest loss negatively impacts biodiversity, affecting ecological (e.g., seed dispersal) and genetic (e.g., genetic diversity and structure) processes. Therefore, understanding how deforestation influences genetic resources is strategic for conservation. Our aim was to empirically evaluate the effects of landscape‐scale forest reduction on the spatial genetic structure and gene flow of *Euterpe edulis* Mart (Arecaceae), a palm tree considered a keystone resource for many vertebrate species. This study was carried out in nine forest remnants in the Atlantic Forest, northeastern Brazil, located in landscapes within a gradient of forest cover (19–83%). We collected leaves of 246 adults and 271 seedlings and performed genotyping using microsatellite markers. Our results showed that the palm populations had low spatial genetic structure, indicating that forest reduction did not influence this genetic parameter for neither seedlings nor adults. However, forest loss decreased the gene flow distance, which may negatively affect the genetic diversity of future generations by increasing the risk of local extinction of this keystone palm. For efficient strategies of genetic variability conservation and maintenance of gene flow in *E. edulis*, we recommend the maintenance of landscapes with intermediary to high levels of forest cover, that is, forest cover above 40%.

## Introduction

Forest loss is the main threat to biodiversity, contributing to the unprecedented levels of the current high rates of species extinction, particularly in tropical regions (Pimm et al. 2014). With the exception of a few wilderness areas, most of extant species in the tropics currently occur in anthropogenic landscapes, where previous continuous forest has been reduced to smaller and increasingly isolated patches. Such change in the amount of remnant forest and the consequent modification of the landscape composition and structure, that is, habitat fragmentation, have important implications for species persistence. Therefore, understanding how such anthropogenic disturbances determine the current biodiversity patterns is a priority for conservation (Keller et al. 2014; Valiente‐Banuet et al. 2014). Such knowledge includes the assessment of genetic diversity, a main component of biodiversity that ultimately encompasses species evolutionary potential (Moritz 2002; Hughes et al. 2008; Laikre et al. 2009).

The structural modifications in anthropogenic landscapes affect genetic diversity within and among forest remnants (Willi and Fischer 2005; Pickup and Young 2008). As landscape‐scale deforestation progresses, mean patch size is reduced and the likelihood of genetic erosion in smaller populations increases due to inbreeding depression and genetic drift; often losses are not offset by migrants as patches become more isolated. Considering that plant dispersers and pollinators are often sensitive to changes in landscape structure (Aizen and Feinsinger 1994; Breed et al. 2013; Morante‐Filho et al. 2015), their mutualistic interactions may also be altered. Therefore, it is expected that tree populations in highly deforested and fragmented landscapes would experience high genetic divergence among isolated populations, reducing gene flow distance (Young et al. 1996) and thus increasing spatial genetic structure (SGS).

Despite such expectations, some widespread tree species are apparently more resilient to such structural changes (Kramer et al. 2008), as they may benefit from long‐distance gene flow via pollen or seeds among forest fragments (Gaiotto et al. 2003; Côrtes et al. 2013). In addition, the *genetic response to recently segregated populations can rather reflect the genetic structure of previously continuous population* (Carvalho et al. 2015). *In particular, due to* the time lag between generations of adults and seedlings (Rigueira et al. 2013), different ontogenetic stages may reflect different time frames in which gene flow was involved, that is, considering some long‐lived trees, the progeny is more likely to reflect recent impacts on genetic structure or gene flow than the adults. Therefore, a comprehensive view on how tree species are impacted by landscape changes should include the assessment of different ontogenetic stages well as different genetic components. For instance, while SGS can be used as an indirect measure of both historical and contemporary gene flow (Vekemans and Hardy 2004), more recent gene flow estimates can be accessed through paternity tests (Ashley 2010; Harrison et al. 2013). Studies with contemporary gene flow approach has shown that forest fragmentation response can vary between species (Kramer et al. 2008) but in general, plants with seeds and pollen dispersed by animals are more sensitive than plants with abiotic dispersion system.

Our aim here was to empirically evaluate the effects of landscape‐scale forest reduction on the spatial genetic structure and the contemporary gene flow of *Euterpe edulis* (Arecaceae). The species is widespread throughout the Brazilian Atlantic Forest (Leitman et al. 2015), a biome currently reduced to 12–16% of its original extension (Ribeiro et al. 2009). This palm is considered a keystone species in Atlantic Forest because it plays an important ecological role, providing food resources for several animal species, including 32 bird and six mammals species (Jordano et al. 2006; Galetti et al. 2013). Its flowers are pollinated by various insects, with the main pollinator being *Trigona spinipes* (Mantovani and Morellato 2000), a small stingless bee with estimated maximum flight distance of <900 m (Zurbuchen et al. 2010). In addition, the appreciation of the apical meristem for human consumption has caused over‐exploitation (Matos et al. 1999) that, in addition to habitat loss, has brought *E. edulis* to the status of endangered species (Martinelli and Moraes 2013).

We conducted surveys in nine forest sites immersed in landscapes varying from 19% to 83% of remaining forest cover with the purpose of test the following hypotheses: (1) Spatial genetic structure is inversely related to the percentage of forest in the landscape for both ontogenetic stages, with a greater effect on seedling populations, reflecting the recent forest loss and fragmentation in the study area; (2) seedling populations in more forested landscapes have fewer assigned parent, reflecting the larger source of pollen and seeds; and (3) landscape‐scale forest reduction negatively influences the gene flow distance, which means that the smallest gene flow distances should be found in more deforested landscapes.

## Materials and Methods

### Study area

**Figure 1:**
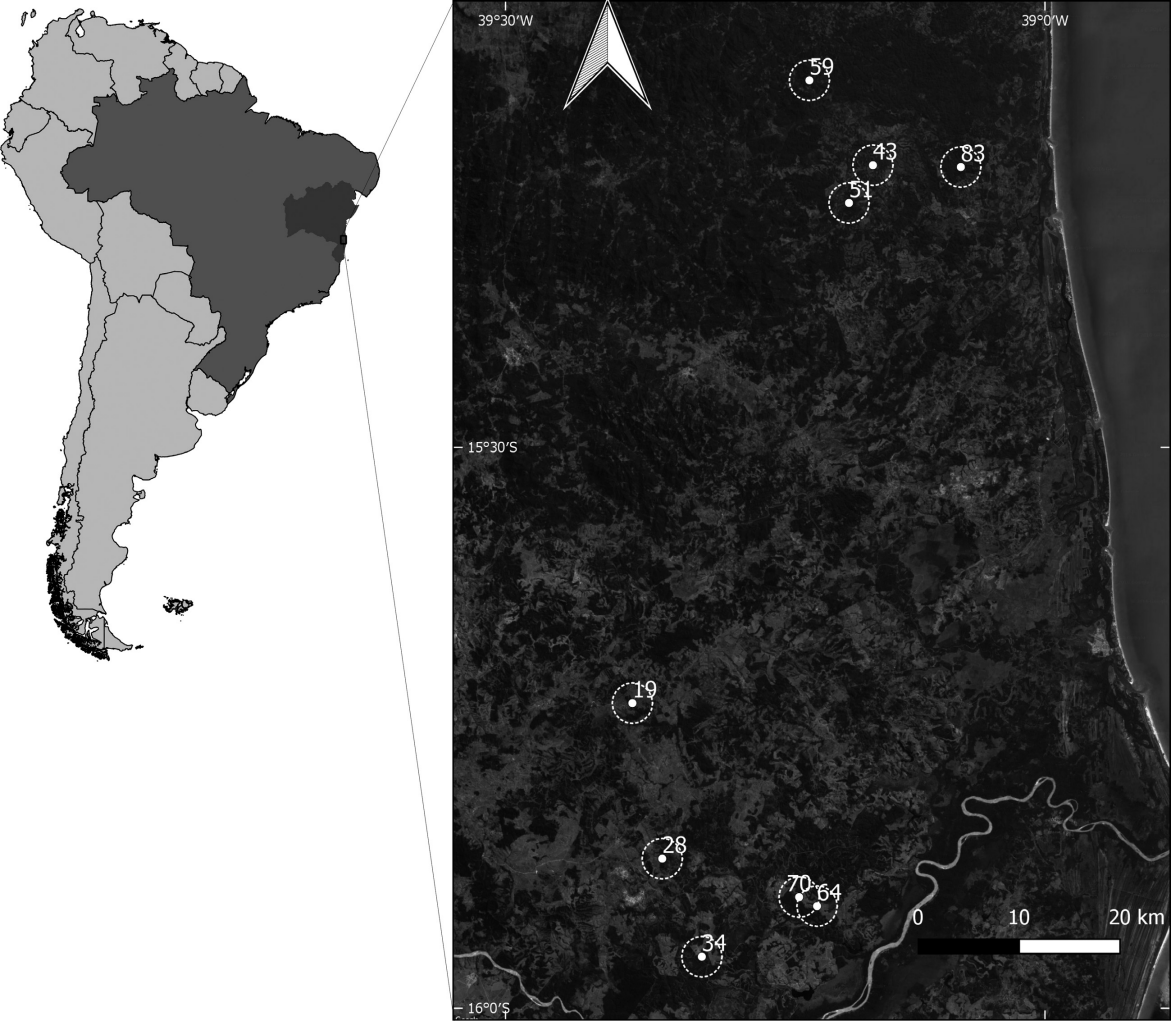
Location of the study region within South America (dark gray: Bahia; light gray: Brazil) and of the study sites within it. White dots indicate the study sites, the circles around them indicate the 2‐km buffers, and the numbers indicate the percentage of forest cover within each buffer. Map data: Google, TerraMetrics, 2016, obtained with the OpenLayers plugin in Quantum GIS 2.14.3 (QGIS Development Team 2016).

The research was conducted in landscapes of the Atlantic Forest in southern Bahia (Fig. 1), where lies the largest forest remnant in northeastern of Brazil (Ribeiro et al. 2009). This region is considered a conservation priority due to its high species richness and endemics (Thomas et al. 1998; Martini et al. 2007). The forest is classified as tropical lowland rainforest (Thomas et al. 1998) with an average annual temperature of 24°C and rainfall of 1.500 mm, with no discernible seasonality (Mori et al. 1983).

### Sampling design

Based on a series of freely available landsat images (1990), we first identified large remaining forest tracts in southern Bahia with similar soil conditions, topography and floristic composition (Thomas et al. 1998), located between the rivers Jequitinhonha and Contas. Using satellite images (QuickBird and WorldView, from 2011 and RapidEye, from 2009 to 2010) and after an intensive ground‐truthing, we elaborated a map of the land use of 3.470 km^2^, which included the municipalities of Belmonte, Una, Santa Luzia, Mascote, and Canavieiras.

This final map was used to identify and visually quantify different categories of land use at the scale of 1:10,000. The forest cover includes not only native forests at different successional stages, but also shade cocoa plantations (*Theobroma cacao*), in addition to rubber and eucalyptus (*Eucalyptus* sp) plantations. However, to estimate forest cover in this study, we considered only the amount of native forest in the landscape, excluding other vegetation classes.

From this map, we identified 58 sites located in forest patches and, using ArcGIS (10.2, QGIS Development Team 2016), we calculated the percentage of native forest in 2 km radius from the central point of site. We selected those sites further than 1 km from each other and from this group we randomly selected 16 sites in landscapes ranging from 6% to 83% of forest cover as sampling sites. In each site, we established a plot of 15 × 400 m for leaf collection from all adults and a plot of 2 × 400 m in the center of the larger one to collect leaves of all seedlings with ≤15 cm in height to evaluate only the recent gene flow, after the forest suppression intensification in the region. This sampling design theoretically would allow us to find the female genitors for most of the sampled seedlings, whereas animals would probably disperse seedlings with no parent attributed. We only considered sites in which at least five individuals of *E. edulis* in each ontogenetic stage were presented within the study plots. Therefore, we included only nine sampling sites with forest cover percentage ranging from 19% to 83% (Fig. 1).

### DNA extraction and genotyping with microsatellite loci

DNA extraction from *E. edulis* leaves was performed using the CTAB protocol (Doyle and Doyle 1987). We genotyped all *E. edulis* individuals, using thirteen microsatellite nuclear loci (Gaiotto et al. 2001). The PCR reactions were performed in multiplex system with the following composition of loci: (1) duplex with EE43 and EE45; EE48 and EE54; EE8, and EE23; and (2) simple PCR with loci: EE32; EE15; EE8, EE3, EE47, EE59, and EE63. Electrophoresis were performed in the capillary system by automated DNA analyzer ABI3500 (Applied Biosystems, Foster City, CA), based on three multiload systems: (1) *pentaload* I, composed of loci EE15, EE32, EE3, EE8, and EE23; (2) *pentaload* II, composed of loci EE43, EE45, EE48, EE54, and EE9; and (3) *triload* composed of loci EE47, EE59, and EE63. Fragment size was estimated with the software GeneMarker version 2.2 (SoftGenetics, State College, PA).

### Data analysis

The spatial genetic structure of *E. edulis* seedlings and adults was estimated separately through the kinship coefficient average (*F*_ij_; Loiselle et al. 1995) in Spagedi version 1.4 (Vekemans and Hardy 2004). To assess the spatial distance between individuals, we attributed eight distance classes with 50 m intervals, between 0 and 400 m. The 95% confidence interval for each distance class was obtained from 10,000 permutations of the individual location. We compared the average spatial genetic structure between the ontogenetic stages of seedlings and adults with a t‐test for independent samples. We also evaluated the average spatial genetic structure of seedlings and adults according to the remaining forest in landscapes by means of simple linear regression analysis. These analyses were performed in R (R Core Team 2016).

We used all seedlings for paternity analysis and each adult as a potential genitor. Paternity was based on statistical Δ (Marshall et al. 1998) and was analyzed in Cervus software version 3.0 (Kalinowski et al. 2007). The critical Δ value for each confidence interval was obtained from 10,000 simulations to determine the most probable parental of unknown sex (father or mother), allowing a proportion of 0.01 of genotyping error per locus, with confidence interval of 95%. We performed a further analysis of paternity exclusion for seedlings whose attributed parents were located at a distance greater than 2 km. The exclusion analysis was performed by comparing the genotypes of the alleged parent against its likely descendant seedling. The paternity index (PI) was estimated for each locus, and the combined paternity index (CPI) between loci was used to infer the paternity probability (PP) according to the method described by Stephenson (2010). We used the same a priori probability of 0.5 used for analysis in humans, where each candidate parent has 50% of chance of being the true parental (Gjertson and Morris 1995). To estimate the PI of each locus, we used the allele frequencies of all loci estimated based on all *E. edulis* adults sampled for this research.

We assessed whether three types of response variables were related to forest cover: (1) the probability of a landscape containing at least one seedling with a parent assigned, as well as with a parent assigned in the same landscape and in another landscape; (2) the proportion of seedlings with assigned parents and that of seedlings with assigned parents in the same landscape and in another landscape; and (3) gene flow distance. For the first analyses, we used generalized linear models (GLMs) with a binomial distribution and using the presence (1) or absence (0) of seedlings with assigned parents (anywhere, in the same landscape and in another landscape) as response variables. For the second analyses, we also used GLMs with a binomial distribution, using the proportion of seedlings with parents assigned in each landscape as response variable and the total number of seedlings as weights to give greater importance to the landscapes that had more seedlings. For the third analyses, we used only the seedlings with assigned parents and applied linear mixed‐effect models (LMMs) between the logarithm of gene flow distance and forest cover, using the landscape identity as a random variable. The logarithms were used to reduce the dispersion of the data.

For each analysis, we calculated significance of the relationship with forest cover in two ways: parametrically, using z‐ratio tests based on the normal distribution for the generalized linear models and t‐ratio tests for the linear mixed models and using Monte Carlo randomizations. For the GLM randomizations, we (1) extracted the deviance of the observed relation between the response variable and habitat cover; (2) randomized the relation between the two variables; (3) performed a GLM on the randomized data set; and (4) extracted the deviance of this GLM. Significance was calculated as the proportion of deviance values in the randomized data set that were equal to or smaller than the observed deviance. When the proportion of seedlings was the response variable, we kept the total number of seedlings associated to each proportion in the randomizations. We assessed the significance of the relation between gene flow and forest cover by (1) performing a simple linear regression between gene flow and forest cover; (2) extracting its *R* ^2^ value; (3) randomly assigning a forest cover (from any of the nine landscapes sampled) to each landscape; (4) performing a linear regression on the randomized data set; and (5) calculating its *R* ^2^. Significance was calculated as the proportion of randomized *R* ^2^ values that were equal to or greater than the observed *R* ^2^. We did not use mixed models in these randomizations because the nonindependence between seedlings located in the same landscape was accounted for by the randomization scheme. In both analyses, we used a total of 4999 randomizations and included the observed deviation in the comparison because it is one of the possible combinations under the null model (Manly 2007). We performed the GLMs, LMMs, and randomizations in R (R Core Team 2016), with the aid of the nlme package (Pinheiro et al. 2016) for the LMM and a code written by us for the randomization tests (available at < https://github.com/pdodonov/MonteCaRlo>).

## Results

**Figure 2:**
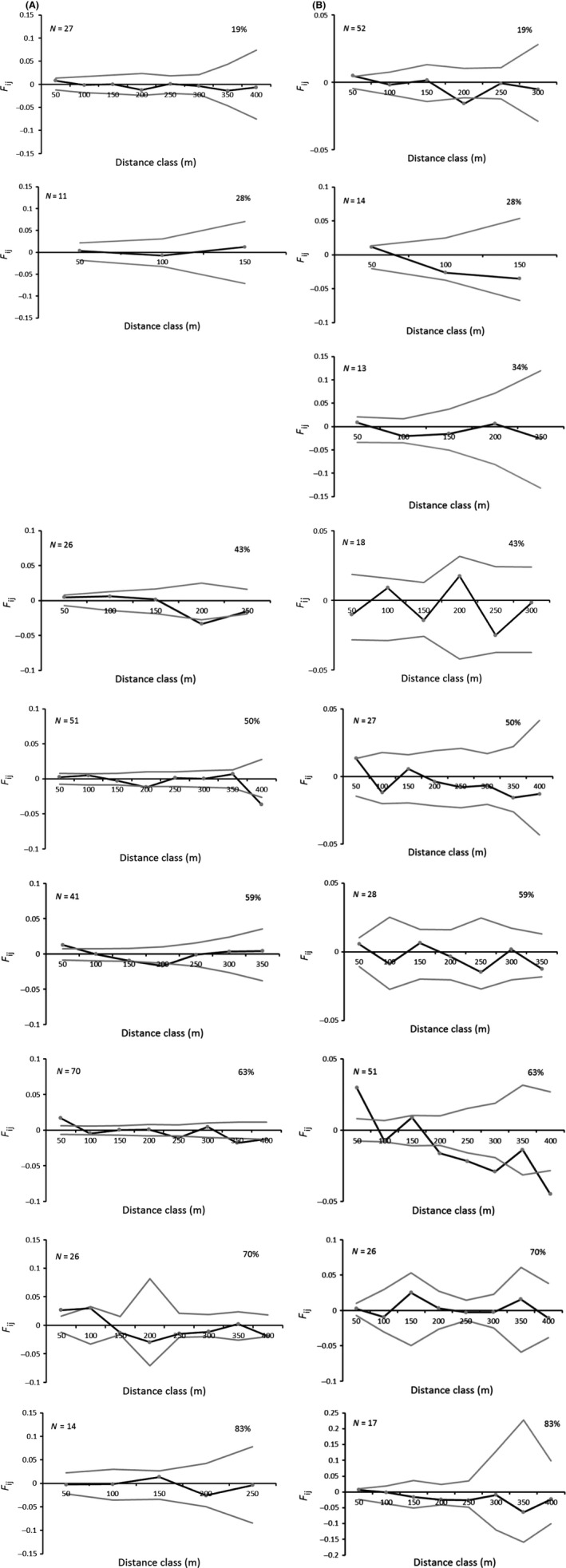
Spatial genetic structure of *Euterpe edulis* (*N* = number of individuals genotyped in each fragment and the respective forest cover, percentage ranging from 19% to 83%). The gray lines indicate 95% confidence interval for average relatedness coefficient (*F*_ij_) observed. (A) *Euterpe edulis* seedlings and (B) adults sampled in nine fragments within landscapes ranging from 19% to 83% of forest.

Landscape‐scale forest loss did not influence the spatial genetic structure of *E. edulis* seedlings and adults (*R* ^2^ = 0.11, *P* = 0.37 and *R* ^2^ = 0.00, *P* = 0.90; respectively). The spatial genetic structure of *E. edulis* populations showed low values of average relatedness coefficient (*F*_ij_) on both ontogenetic stages and they were not related to distance class (Fig. 2A and B). Regarding the seedling population located in the forest site within 34% of landscape‐scale forest cover, we recorded only five individuals, making impossible the construction of the SGS figure, as the average relatedness coefficient (*F*_ij_) observed (0.0138) coincides with the minimum and maximum values of the confidence interval. The seedlings' average spatial genetic structure value is low (0.001), but significantly higher (*P* = 0.034) than the adults' average value (−0.003).

**Figure 3:**
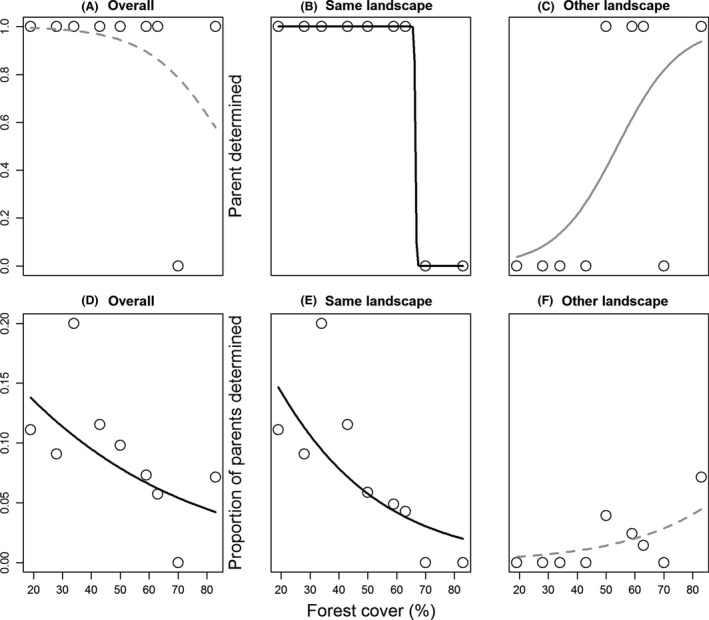
Presence (A–C) and proportion (D–F) of seedlings with assigned parents overall (A, D), in the same landscape (B, E), and in another landscape (C, F) as related to forest cover in the landscape. The lines represent generalized linear models with a binomial distribution. Line type represents significance as assessed by Monte Carlo tests: *P* ≤ 0.05 (full black line), 0.05 < *P* ≤ 0.10 (full gray line), *P* > 0.10 (dashed gray line).

Of the 271 seedlings analyzed, we assigned the parent (father/mother) to 21 individuals (7.75%; Table 1). Of these 21 individuals, 16 were assigned a parent within the same landscape (2 km radius) and five seedlings were assigned a parent located in another landscape (Table 1). The probability of a landscape having at least one seedling with a parent assigned was not related to forest cover (parametric *P *=* *0.34, Monte Carlo *P *=* *0.44; Fig. 3A). However, seedlings with assigned parents in the same landscape were more likely to be found in lower forest cover (Monte Carlo *P* = 0.03; Fig. 3B. No parametric p was available due to a perfect separation in the response variable) whereas those with parents in other landscapes were marginally more likely to occur in higher forest cover (parametric *P* = 0.12, Monte Carlo *P* = 0.08). The proportion of seedlings with an assigned showed decreased as forest cover increased (parametric *P* = 0.12, Monte Carlo *P* = 0.03; Fig. 3D). The proportion of seedlings with an assigned parent in the same landscape also decreased as forest cover increased (parametric *P* = 0.02, Monte Carlo *P* = 0.002; Fig. 3E) whereas the proportion of seedlings with an assigned parent in another landscape was not related to forest cover (parametric *P* = 0.29, Monte Carlo *P* = 0.18; Fig. 3F).

**Table 1:** Number of individuals of *Euterpe edulis* adults and seedlings and number seedlings with an assigned parent (father or mother) sampled in nine fragments immersed in landscapes in the Atlantic Forest of southern Bahia, Brazil, varying from 19% to 83% forest cover

%^a^	Adults	Seedlings	Seedlings with parent within the same landscape	Seedlings with parent within another landscape	Number of seedlings with parent
19	52	27	3	0	3
28	14	11	1	0	1
34	13	5	1	0	1
43	18	26	3	0	3
50	27	51	3	2	5
59	28	41	2	1	3
63	51	70	3	1	4
70	26	26	0	0	0
83	17	14	0	1	1
Overall	246	271	16	5	21

a Forest cover percentage in 2 km radius around sampling site in landscapes.

**Figure 4:**
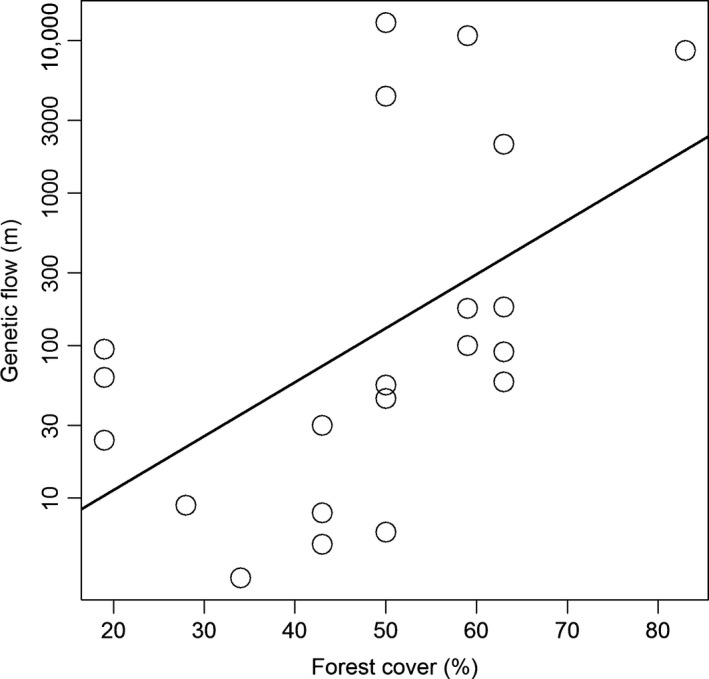
Gene flow distance of each seedling with an assigned parent as related to forest cover. The line represents the fit of a linear mixed model performed on the logarithm of gene flow distance and forest cover, with landscape included as random factor (*P* < 0.05). Please note that the *y*‐axis is on a logarithmic scale.

*Euterpe edulis* geographic distance of gene flow increased with forest cover (Parametric *P* = 0.04, Monte Carlo *P* = 0.02; Fig. 4). In addition, the highest distances of gene flow were only recorded in the more forested landscapes. Furthermore, our results demonstrate that long‐distance gene flow (maximum distance of 13 km) only occurs between populations located in intermediate and high percentage of forest cover (≥43%; Table 2).

**Table 2:** Gene flow between landscapes considering the landscape forest percentage of progeny (%^1^); landscape forest percentage of parent (%^2^); probability paternity or maternity obtained using a priori probability of 0.5 (*P* = 0.5); distance (km) is the geographic distance between the landscapes

Progeny	%^1^	Parent	%^2^	*P* = 0.5	Distance (km)
P172	63	A185	70	0.998672931	2
P061	50	A003	43	0.999930484	5
P275	83	A018	43	0.998812611	8
P106	59	A016	43	0.999767368	10
P055	50	A080	59	0.999223400	13

## Discussion

In this study, we did not find evidence that landscape‐scale deforestation influenced the spatial genetic structure (SGS) of *E. edulis* populations in our study region. Furthermore, we showed an increase in seedlings with parental assignment and a decrease in gene flow distance in more deforested landscapes. The negative effects of habitat loss have been previously shown in theoretical and empirical studies on biodiversity patterns (Andrén 1994; Fahrig 2002), including ecological interactions (Valiente‐Banuet et al. 2014). We emphasize that a previous study has revealed a likely time lag between landscape change and genetic diversity loss in populations of *E. edulis* in the same study areas in southern Bahia (Santos et al. 2015). Now we have indication that the gene flow is the genetic parameter that first experiences the negative influence of such anthropogenic disturbances, measured as landscape‐scale forest loss. Therefore, although the *E. edulis* populations evaluated are still harboring high levels of genetic diversity as described by Santos et al. (2015), such populations are experiencing a reduction in contemporary gene flow.

Our results showed that kinship values did not decrease with geographic distance as expected from the isolation‐by‐distance model (Wright 1943; Barbujani 1987). The SGS in *E. edulis* seedling and adult populations is considered low for plant species with pollen and seed dispersal over long distances (Loiselle et al. 1995). The low values of the average relatedness coefficient (*F*_ij_) in both ontogenetic stages indicate that individuals are not closely related (low kinship levels) (Hamrick and Trapnell 2011). Such low SGS converges with the previous result reporting high genetic variability within these same populations of *E. edulis* (Santos et al. 2015). In addition, the low levels of SGS found in *E. edulis* seedling populations within a gradient of deforestation may occur due to different ecological processes. In deforested landscapes, for instance, we expected a limitation in food resources (Fahrig 2003) to make the few seed dispersers that are still present (Morante‐Filho et al. 2015) move longer distances in search of the scarce resources. In this situation, the seeds could be dispersed farther from the parent, resulting in low levels of kinship between nearby individuals, reflecting the low SGS in landscapes with low percentage of forest. By contrast, in more forested landscapes, the higher levels of richness and abundance of seed dispersers (Morante‐Filho et al. 2015) could increase the rates of long‐distance dispersal, also resulting in a low level of relatedness and SGS. The aforementioned dispersion behavior associated with the naturally high genetic diversity of individuals contributing to pollen and seeds can explain the absence of SGS found in all the analyzed landscapes. The low SGS found in adult populations may reflect older ecological processes influenced by the genetic structure and diversity of past generations, when landscapes contained more continuous forests (Aguilar et al. 2008; Metzger et al. 2009; Landguth et al. 2010). Furthermore, the average SGS value for seedling populations was significantly higher than the average SGS for adult populations. Considering that the mean values of the relatedness of seedlings (0.001) and adults (−0.003) are low and very close to zero, the biological interpretation points to a similarity between both ontogenetic stages. Indeed, such low values were expected as *E. edulis*, being a tree species, would require a long time period to accumulate genetic changes (Landguth et al. 2010). In addition, land‐use change and mainly deforestation in the region occurred less than 50 years prior to the study (after 1960 according to Alger and Caldas 1994), and probably, there was no enough time for a significant genetic change in *E. edulis* populations.

The low percentage of seedlings with an assigned parent indicates that, notwithstanding the patterns of forest loss and fragmentation, dispersal of pollen and seeds is still occurring within and between landscapes. Moreover, although forest loss may not affect the density of individuals (Santos et al. 2015), we found a lower proportion of seedlings with an assigned parent in more forested landscapes, indicating that gene flow is occurring outside our sample plot. However, in landscapes with less than 19% of forest cover, we found less than five individuals of *E. edulis*. With such low abundances, the long‐distance gene flow does not occur and we expect an increase in population vulnerability due to stochastic factors. Therefore, our results suggest that an increase in kinship between individuals can occur in a near future in the more deforested landscapes. As a consequence, we predict an increase in inbreeding and a reduction in genetic diversity over the generations (Quesada et al. 2013) that in turn would influence the long‐term viability of populations in deforested landscapes.

We detected that the geographic distance of gene flow increased with forest cover. In addition, the highest distances of gene flow were only recorded in the more forested landscapes. The shortest distance of gene flow found in deforested landscapes likely indicates a limitation of seed dispersal agents (Scheepens et al. 2012) or lower availability pollen and seed donors compared with forested landscapes. It is known that the richness of frugivorous birds, which are the main dispersers of *E. edulis* seeds in the region, abruptly decreases as deforestation progress (Morante‐Filho et al. 2015). A decrease in seed dispersion distances is therefore also expected, directly influencing the reduction of gene flow in these landscapes. Furthermore, pollinators in deforested landscapes may occur in smaller number and be less effective than in forested landscapes (Dáttilo et al. 2015), representing a possible negative influence on the species gene flow distances and possibly driving related processes on the local scale (Breed et al. 2013).

Our results showed contemporary gene flow with a maximum distance of 13 km between landscapes. Long‐distance dispersal is critical for species persistence, especially considering anthropogenic disturbances such as habitat modification (Trakhtenbrot et al. 2005) and climate change (McCallum et al. 2014). Thus, due to the critical reduction and fragmentation of the Atlantic Forest (Ribeiro et al. 2009), potential gene flow over long distances is vital for *E. edulis* persistence. The sporadic gene flow among forest remnants may contribute to population dynamics maintenance, minimizing or inhibiting genetic differentiation between populations and preventing a possible reduction of local genetic diversity (Hardesty et al. 2006; Kramer et al. 2008). Therefore, gene flow over long distances is important for maintaining functional (genetic) connection among populations (Matter et al. 2013; Colabella et al. 2014), minimizing the chances of inbreeding and genetic drift (McCallum et al. 2014). Hence, the knowledge of long‐distance gene flow allows to propose efficient strategies for ecological connectivity (Carlo et al. 2013).

Our data did not allow us to detect whether the observed long‐distance gene flow occurred via pollen or seed. However, considering a previous record of *E. edulis* gene flow of 22 km that was attributed to seed dispersal (Gaiotto et al. 2003), we can assume that gene flow found in this study is probably due to seed dispersal by birds, which are the main seed dispersers of *E. edulis* (Galetti et al. 2013). Furthermore, the main pollinator (i.e., the bee species *Trigona spinipes*) has short displacement distance (Roubik & Aluja 1983; Araujo et al. 2004; Zurbuchen et al. 2010). Additionally, the contemporary gene flow results converging with results from F_ST_ analyzes (Santos et al. 2015) and SGS. Our results indicated low spatial genetic structure that is characteristic of species with long‐distance dispersal, and little genetic differentiation among populations (*F*_ST_), probably an indication of high rates of gene flow.

The long‐distance gene flow was recorded only between landscapes with intermediate and high forest coverage (≥43%). This result may reflect the greater richness of frugivorous birds in these landscapes compared with little‐forested landscapes (Morante‐Filho et al. 2015) as well as higher connectivity among fragments, allowing both seed dispersal and short‐distance pollination. Another plausible hypothesis is that, in deforested landscapes, few suitable habitats for seed germination and seedling establishment are found. Assuming that seeds can reach different sites in our deforested landscapes, recruitment is limited by unsuitable local conditions (Lowe et al. 2005). In such situations, populations in deforested landscapes are more likely to increase the degree of genetic differences in relation to other populations, and changes would occur in the gene pool over time (Santos et al. 2015).

In conclusion, for landscape‐level conservation strategies aimed to maintain micro‐evolutionary processes through gene flow, we recommend avoiding decrease in forest cover below 40%. Based on *E. edulis* genetic analysis, our recommendation for conservation was similar to that reported for other populations of the same species in Southeastern Brazil (Carvalho et al. 2015). However, due to the older history of fragmentation in Brazilian southeastern landscapes (Carvalho et al. 2015), the effects of forest loss on genetic parameters there are much more evident than in southern Bahia (northeast Brazil). Thus, we can predict that, with time, the negative effects of forest loss in southern Bahia will be similar to those found in Southeastern Brazil (São Paulo State), an example that reinforces the time lag of tropical forests. Conversely, landscapes with forest cover below this threshold value should be managed to maintain genetic diversity (Santos et al. 2015) and intrapopulation gene flow. This recommendation provides time for long‐term conservation strategies (e.g., landscape management, habitat restoration). Our study contributes to the understanding of functional connectivity between landscapes with different percentages of remaining forest. Furthermore, our results provide an innovative character of relevance to unravel the impacts generated by the landscape‐scale deforestation in SGS and contemporary gene flow to one plant species of the rainforest. Moreover, our findings may aid in assisting an efficient management and conservation of *E. edulis* (Keller et al. 2014)*,* a keystone species highly threatened by harvesting and habitat modification.

## Data Accessibility

http://datadryad.org/

## Conflict of Interest

The authors declare no conflict of interests.
